# Supplementary material for: The greening-causing agent alters the behavioral and electrophysiological responses of the Asian citrus psyllid to a putative sex pheromone
Source: Sci Rep. 2024 Jan 3;14:455. doi: 10.1038/s41598-023-50983-8 (PMC10764743; doi:10.1038/s41598-023-50983-8)
Supplement: Supplementary file 1 — Supplementary Information. [file 41598_2023_50983_MOESM1_ESM.pdf]

Raw data for Figure 1 (2 pages)

2/18	2/20	3/9	3/13	3/16	29/04	5/4	5/9	5/13	5/18	5/23	6/2	6/6
1	0.54	1	1.5	1.22	0.82	0.54	0.67	1.22	0.54	0.67	0.33	0.54
0.43	0.4	1.5	0.67	0.43	0.82	0.33	0.67	0.25	0.67	0.43	0.54	0.54
1	0.54	1.22	0.82	0.67	1.5	1.5	0.33	0.33	0.67	1.22	0.67	0.67
0.11	1	1.22	0.54	0.82	0.33	0.67	0.54	0.67	0.82	0.43	0.67	0.54
0.38	0.5	0.54	0.54	0.43	0.82	1	0.54	0.54	1.5	1.22	0.31	0.67

6/13	6/15	6/26	07/07	7/10	8/28	9/18	9/25	10/9	10/22	11/27	12/22
0.43	0.67	0.33	0.54	0.33	1.1	0.67	0.82	0.43	0.67	1.5	0.82
0.43	0.33	0.33	1	0.18	0.54	0.82	1.5	1.5	1.22	1.22	0.33
0.33	0.54	0.25	0.67	0.25	0.31	0.43	0.43	0.67	1.22	1.22	0.18
0.33	0.82	0.54	1	0.33	0.31	0.25	0.67	0.5	1	0.67	1
0.18	0.67	0.25	0.18	0.43	1	0.67	0.82	1.22	0.54	1.22	0.67

Raw data for Figure 2

Male	Female	Male	Female
96.77	100	100	100
100	100	100	100
100	97.96	100	100
100	100	100	98.25
100	100	97.44	100
100	100	93	100
100	100	100	94.9
100	100	100	100
100	100	90.8	97.1
100	100	95.7	100
100	98.2	98.2	100

Raw data for Figure 3 (3 pages)

Figure 3A		Figure 3B		Figure 3C		Figure 3D	
**AA-1 µg**	**C**	**AA-1 µg**	**C**	**AA-10 µg**	**C**	**AA-50 µg**	**C**
10	0	5.56	4.44	5.08	4.92	10	0
8.1	1.9	4.1	5.9	6.97	3.03	10	0
1.99	8.01	10	0	9.46	0.54	0	10
4.13	5.87	2.05	7.95	0	10	10	0
8.54	1.46	6.2	3.8	6.83	3.17	6.89	3.11
10	0	4.28	5.72	4	6	0	10
0	10	6.06	3.94	4.47	5.53	9.09	0.91
4.14	5.86	0	10	1.86	8.14	7.34	2.66
6.57	3.43	2.56	7.44	8.16	1.84	0	10
1.94	8.06	0	10	10	0	6.46	3.54
1.66	8.34	0	10	10	0	1.1	8.9
3.88	6.12	5.98	4.02	6.88	3.12	8.05	1.95
1.32	8.68	5.45	4.55	2.87	7.13	9.78	0.22
7.29	2.71	9.12	0.88	5.18	4.82	8.27	1.73
6.29	3.71	7.53	2.47	1.8	8.2	8.27	1.73
6.9	3.1	9.76	0.24	9.39	0.61	6.01	3.99
10	0	10	0	6.45	3.55	7.7	2.3
8.33	1.67	10	0	7.12	2.88	4	6
3.87	6.13	5.17	4.83	0	10	9.65	0.35
8.12	1.88	6.65	3.35	1.56	8.44	7.06	2.94
6.44	3.56	7.06	2.94	8.84	1.16	5.12	4.88
0	10	0.97	9.03	10	0	0	10
6.71	3.29	6	4	3.1	6.9	8.91	1.09
0	10	10	0	0.34	9.66	7.68	2.32
9.43	0.57	8.24	1.76	10	0	10	0
0	10	3.21	6.79	0	10	7.89	2.11
7.04	2.96	4.54	5.46	10	0	3.07	6.93
10	0	5.89	4.11	10	0	0	10
8.03	1.97	4.54	5.46	0.9	9.1	7.75	2.25
7.87	2.13	3.73	6.27	2.34	7.66	10	0
8.62	1.38	4.48	5.52	6.6	3.4	3.85	6.15
3.3	6.7	1.81	8.19	8.02	1.98	5.82	4.18
8.99	1.01	8.2	1.8	3.97	6.03	0	10
7	3	2.71	7.29	5.4	4.6	10	0
6.28	3.72	8.19	1.81	6.88	3.12	1.64	8.36
10	0	3.89	6.11	2.82	7.18	10	0
6.93	3.07	9.09	0.91	7.06	2.94	9.54	0.46
9.91	0.09	10	0	5.87	4.13	10	0
7.37	2.63	5.66	4.34	6.2	3.8	2.29	7.71
5.3	4.7	7.08	2.92	1.12	8.88	5.85	4.15
0	10	0.95	9.05	7.45	2.55	0	10
7.85	2.15	3.53	6.47	8.86	1.14	6.06	3.94

5.04	4.96	0	10	6.14	3.86	8.07	1.93
2.29	7.71	2.03	7.97	1.03	8.97	8.81	1.19
4.01	5.99	4.3	5.7	6.02	3.98	6.62	3.38
7.82	2.18	4.3	5.7	2.07	7.93	10	0
2.8	7.2	4.01	5.99	1.32	8.68	9.72	0.28
9.3	0.7	6.16	3.84	4.02	5.98	8.63	1.37
4.31	5.69	2.12	7.88	8.19	1.81	6.65	3.35
3.96	6.04	10	0	4.05	5.95	1.27	8.73
9.72	0.28	3.96	6.04	6.06	3.94	9.07	0.93
8.22	1.78	9.01	0.99	1.22	8.78	0	10
10	0	6.83	3.17	10	0		
0	10	0	10	10	0		
6.22	3.78	0	10	10	0		
9.31	0.69	6.93	3.07	2.81	7.19		
5.05	4.95	6.43	3.57	3.07	6.93		
7.42	2.58	9.67	0.33	1.7	8.3		
6.72	3.28			3.3	6.7		
4.4	5.6			6.23	3.77		
6.27	3.73			7.56	2.44		
3.78	6.22			9.22	0.78		
0	10			4.66	5.34		
8.97	1.03			2.28	7.72		
0.1	9.9			0	10		
4.96	5.04			4.13	5.87		
8.05	1.95						
2.85	7.15						
3.43	6.57						
4.97	5.03						
6.17	3.83						
4.23	5.77						
10	0						
8.35	1.65						
4.06	5.94						
5.57	4.43						
3.01	6.99						
7.19	2.81						
8.21	1.79						
7.64	2.36						
10	0						
7.97	2.03						
5.15	4.85						
6.99	3.01						
2.07	7.93						
1.22	8.78						
8.36	1.64						

8.36	1.64
0	10
3.02	6.98
8.75	1.25
7.37	2.63
6.04	3.96
7.77	2.23

Raw data for Figure 4

Clas-free 1	Clas+ 1	Clas-free 50	Clas+ 50
0.931	1.48	1.74	6.256
0.382	5.981	6.668	13.946
1.907	2.121	3.189	16.8
0.687	4.456	4.166	16.525
0.656	2.014	2.793	10.437
1.007	1.267	5.051	12.894
